# Supplementary material for: A new synonym for Viburnum erosum (Viburnaceae) in East China, based on morphological and molecular evidence
Source: PLoS One. 2025 Apr 23;20(4):e0312920. doi: 10.1371/journal.pone.0312920 (PMC12017528; doi:10.1371/journal.pone.0312920)

PLOS ONE

A new synonym for Viburnum erosum (Viburnaceae) in East China, based on morphological and molecular evidence

--Manuscript Draft--

**Manuscript Number:**	PONE-D-24-45964
**Article Type:**	Research Article
**Full Title:**	A new synonym for Viburnum erosum (Viburnaceae) in East China, based on morphological and molecular evidence
**Short Title:**	A new synonym for Viburnum erosum, based on morphological and molecular evidence
**Corresponding Author:**	Ming Tang, Ph.D.<br>Jiangxi Agricultural University<br>Nanchang, Jiangxi CHINA
**Keywords:**	Viburnaceae, Viburnum, synonym, taxonomy, China
**Abstract:**	The critical observations of living plants in the field, along with the examination of type specimens and protologues, led us to conclude that the key characteristics, including the length of the petiole, stipules and peduncles, as well as the shape of leaves of Viburnum fengyangshanense, all fall within the variation range of V. erosum. Additionally, molecular analysis of nuclear ribosomal internal transcribed spacer (nrITS) and three plastid DNA markers (rbcL, matK and ndhF) indicates that V. fengyangshanense and V. erosum are deeply nested within a clade. Therefore, based on morphological and molecular evidence, it is demonstrated that V. fengyangshanense should be regarded as a new synonym of V. erosum.
**Order of Authors:**	Liaocheng Zhao
	Ming Tang, Ph.D.
	Yitian Wang
	Wenjun Lyu
	Zijian Tang
	Lihong Qiu
**Additional Information:**	
**Question**	**Response**
**Financial Disclosure**<br><br>Enter a financial disclosure statement that describes the sources of funding for the work included in this submission. Review the submission guidelines for detailed requirements. View published research articles from *PLOS ONE* for specific examples.<br><br>This statement is required for submission and **will appear in the published article** if the submission is accepted. Please make sure it is accurate.	Yes

Powered by Editorial Manager® and ProduXion Manager® from Aries Systems Corporation

**Funded studies**<br>Enter a statement with the following details:<br>• Initials of the authors who received each award<br>• Grant numbers awarded to each author<br>• The full name of each funder<br>• URL of each funder website<br>• Did the sponsors or funders play any role in the study design, data collection and analysis, decision to publish, or preparation of the manuscript?<br><br>Did you receive funding for this work?	
Please add funding details.<br>as follow-up to "**Financial Disclosure**<br><br>Enter a financial disclosure statement that describes the sources of funding for the work included in this submission. Review the submission guidelines for detailed requirements. View published research articles from *PLOS ONE* for specific examples.<br><br>This statement is required for submission and **will appear in the published article** if the submission is accepted. Please make sure it is accurate.<br><br>**Funded studies**<br>Enter a statement with the following details:<br>• Initials of the authors who received each award<br>• Grant numbers awarded to each author<br>• The full name of each funder<br>• URL of each funder website<br>• Did the sponsors or funders play any role in the study design, data collection and analysis, decision to publish, or preparation of the manuscript?<br><br>Did you receive funding for this work?"	National Natural Science Foundation of China (grant no. 31960043)
Please select the country of your main research funder (please select carefully as in some cases this is used in fee calculation).	CHINA - CN

as follow-up to "**Financial Disclosure**<br><br>Enter a financial disclosure statement that describes the sources of funding for the work included in this submission. Review the submission guidelines for detailed requirements. View published research articles from *PLOS ONE* for specific examples.<br><br>This statement is required for submission and **will appear in the published article** if the submission is accepted. Please make sure it is accurate.<br><br>**Funded studies**<br>Enter a statement with the following details:<br>• Initials of the authors who received each award<br>• Grant numbers awarded to each author<br>• The full name of each funder<br>• URL of each funder website<br>• Did the sponsors or funders play any role in the study design, data collection and analysis, decision to publish, or preparation of the manuscript?<br><br>Did you receive funding for this work?"	
**Competing Interests**<br><br>Use the instructions below to enter a competing interest statement for this submission. On behalf of all authors, disclose any competing interests that could be perceived to bias this work—acknowledging all financial support and any other relevant financial or non-financial competing interests.<br><br>This statement is required for submission and **will appear in the published article** if the submission is accepted. Please make sure it is accurate and that any funding sources listed in your Funding Information later in the submission form are also declared in your Financial Disclosure statement.	The authors have declared that no competing interests exist.

View published research articles from [*PLOS ONE*] for specific examples.<br><br>**NO authors have competing interests**<br><br>Enter: *The authors have declared that no competing interests exist.*<br><br>**Authors with competing interests**<br><br>Enter competing interest details beginning with this statement:<br><br>*I have read the journal's policy and the authors of this manuscript have the following competing interests: [insert competing interests here]*<br><br>* typeset	
**Ethics Statement**<br><br>Enter an ethics statement for this submission. This statement is required if the study involved:<br><br>• Human participants<br>• Human specimens or tissue<br>• Vertebrate animals or cephalopods<br>• Vertebrate embryos or tissues<br>• Field research<br><br>Write "N/A" if the submission does not require an ethics statement.<br><br>General guidance is provided below. Consult the submission guidelines for detailed instructions. **Make sure that all information entered here is included in the Methods section of the manuscript.**	N/A

Powered by Editorial Manager® and ProduXion Manager® from Aries Systems Corporation

**Format for specific study types**<br><br>**Human Subject Research (involving human participants and/or tissue)**<br>• Give the name of the institutional review board or ethics committee that approved the study<br>• Include the approval number and/or a statement indicating approval of this research<br>• Indicate the form of consent obtained (written/oral) or the reason that consent was not obtained (e.g. the data were analyzed anonymously)<br><br>**Animal Research (involving vertebrate animals, embryos or tissues)**<br>• Provide the name of the Institutional Animal Care and Use Committee (IACUC) or other relevant ethics board that reviewed the study protocol, and indicate whether they approved this research or granted a formal waiver of ethical approval<br>• Include an approval number if one was obtained<br>• If the study involved *non-human primates*, add *additional details* about animal welfare and steps taken to ameliorate suffering<br>• If anesthesia, euthanasia, or any kind of animal sacrifice is part of the study, include briefly which substances and/or methods were applied<br><br>**Field Research**<br><br>Include the following details if this study involves the collection of plant, animal, or other materials from a natural setting:<br>• Field permit number<br>• Name of the institution or relevant body that granted permission	
**Data Availability**<br><br>Authors are required to make all data underlying the findings described fully available, without restriction, and from the time of publication. PLOS allows rare exceptions to address legal and ethical concerns. See the PLOS Data Policy and FAQ for detailed information.	Yes - all data are fully available without restriction

Powered by Editorial Manager® and ProduXion Manager® from Aries Systems Corporation

A Data Availability Statement describing where the data can be found is required at submission. Your answers to this question constitute the Data Availability Statement and **will be published in the article**, if accepted.<br><br>**Important:** Stating 'data available on request from the author' is not sufficient. If your data are only available upon request, select 'No' for the first question and explain your exceptional situation in the text box.<br><br>Do the authors confirm that all data underlying the findings described in their manuscript are fully available without restriction?	
**Describe where the data may be found in full sentences. If you are copying our sample text, replace any instances of XXX with the appropriate details.**<br><br>• If the data are **held or will be held in a public repository**, include URLs, accession numbers or DOIs. If this information will only be available after acceptance, indicate this by ticking the box below. For example: *All XXX files are available from the XXX database (accession number(s) XXX, XXX.)*.<br>• If the data are all contained **within the manuscript and/or Supporting Information files**, enter the following: *All relevant data are within the manuscript and its Supporting Information files.*<br>• If neither of these applies but you are able to provide **details of access elsewhere**, with or without limitations, please do so. For example:<br><br>*Data cannot be shared publicly because of [XXX]. Data are available from the XXX Institutional Data Access / Ethics Committee (contact via XXX) for researchers who meet the criteria for access to confidential data.*<br><br>*The data underlying the results presented in the study are available from (include the name of the third party*	All relevant data are within the manuscript and its Supporting Information files.

and contact information or URL).<br>• This text is appropriate if the data are owned by a third party and authors do not have permission to share the data.<br><br>* typeset	
Additional data availability information:	Tick here if your circumstances are not covered by the questions above and you need the journal’s help to make your data available.

Manuscript

Click here to access/download;Manuscript;A new synonym for Viburnum erosum.docx

A new synonym for *Viburnum erosum* (Viburnaceae) in East China, based on morphological and molecular evidence

Liaocheng Zhao[1,2], Yitian Wang[2], Wenjun Lyu[3], Zijian Tang[2], Lihong Qiu [2, 4*], Ming Tang [2, 4*]

1 Laboratory of Systematic Evolution and Biogeography of Woody Plants, College of Ecology and Nature Conservation, Beijing Forestry University, Beijing, China

2 College of Forestry, Jiangxi Agricultural University, Nanchang, Jiangxi, China

3 National Germplasm Repository of *Viburnum*, Wuhan Botanical Garden, Chinese Academy of Sciences, Wuhan, Hubei, China

4 Jiangxi Provincial Key Laboratory of Conservation Biology (2023SSY02081), Jiangxi Agricultural University, Nanchang, Jiangxi, China

*tangming@jxau.edu.cn (MT); qiulihong@jxau.edu.cn (LQ)

Abstract

The critical observations of living plants in the field, along with the examination of type specimens and protologues, led us to conclude that the key characteristics, including the length of the petiole, stipules and peduncles, as well as the shape of leaves of *Viburnum fengyangshanense*, all fall within the variation range of *V. erosum*. Additionally, molecular analysis of nuclear ribosomal internal transcribed spacer (nrITS) and three plastid DNA markers (*rbcL*, *matK* and *ndhF*) indicates that *V. fengyangshanense* and *V. erosum* are deeply nested within a clade. Therefore, based on morphological and molecular evidence, it is demonstrated that *V. fengyangshanense* should be regarded as a new synonym of *V. erosum*.

Keywords

Viburnaceae, *Viburnum*, synonym, taxonomy, China

Introduction

Viburnum Linnaues [1] comprises approximately two hundred species of shrubs and small trees worldwide, distributed from the subtropical northern hemisphere to the Andes Mountain and tropical Asia. China is one of the core distribution centers of this genus with 73 species (45 endemics) [2]. *Viburnum* was originally classified within Caprifoliaceae [3-4]. Phylogenetic studies based on morphological traits and molecular data indicate that *Viburnum* differs from other genera in Caprifoliaceae and has a close genetic relationship with Adoxaceae, which includes *Adoxa* Linnaeus, *Tetradoxa* C.Y. Wu, and *Sinadoxa* C.Y. Wu, Z.L. Wu & R.F. Huang [5]. This classification has been accepted in the taxonomic filed [2, 6-7]. However, some scholars have proposed that the scientific name of this family should be Viburnaceae. Reveal [8] suggested that if *Viburnum* is to be independently established as Viburnaceae, the names Viburnaceae should be conserved, and Adoxaceae should be "superconserved" when Viburnaceae merges with the latter. The ICN [9] approved the retention of Viburnaceae, but the proposal to conserve Adoxaceae was rejected. The adoption of these recommendations indicates that Viburnaceae should be recognized as the scientific name for a new taxa formed together with *Viburnum*, *Sambucus* Linnaeus, *Tetradoxa*, *Sinadoxa* and *Adoxa*.

Viburnum erosum Thunberg [10] was first collected by Carl Peter Thunberg during his botanical exploration in Japan, but the species was initially described and published by Johan Andreas Murray [10] based on *C. P. Thunberg s.n.* (UPS) (Fig. 1A) from Japan without specifying a precise locality. Due to its considerable variability in various morphological features, such as petiole and stipule length, and particularly the density of indumentum on stems, abaxial leaves and peduncles [2, 4, 11-12], serveral names, including *V. erosum* var. *atratocarpum* Hsu [11] (Fig. 2A), *V. erosum* var. *ichangense* Hemsley [13] (Fig. 2B), *V. erosum* var. *setchuenense* Graebner [14] (Fig. 2C), *V. taquetii* H. Léveillé [15] (Fig. 2D) were initially published as new taxa but later considered as synonyms of *V. erosum* (Fig. 1A) for their traits falling within the variation range of the latter [2, 4, 11]. In addition, *V. erosum* represents a taxonomic complex with significant morphological variations,

and widely distributed across most provinces of China and other Asian regions, such as South Korea, North Korea and Japan (Fig. 6).

Viburnum fengyangshanense Z.H. Chen, P.L. Chiu, et L.X. Ye [16] was first described based on *Z. H. Chen et al. LQ2016001* (holotype, ZM; isotype, HTC, ZM; color plate Fig. 1B) collected in bush and slope nearby Fengyang Lake in Fengyang Mountain, Longquan County, Zhejiang, China. Qiu noted in the protologue that this species is distinct from other *Viburnum* species for its longer stipules and bigger leaves [16]. He also claimed that it resembles *V. erousm* in certain quantitative traits, such as larger leaves and fruits, and longer peduncles, stipules, petiole, but he did not provide type specimens of this species nor conduct molecular biology study.

During our filed work in May 2023 in Zhejiang, China, we discovered a unique *Viburnum* population in Fengyang Mountain, Longquan County. These plants are 1-2 m tall, with compound umbel-like cyme featuring a 1.5-3 cm peduncle, ovate-elliptic leaves, lateral veins 6-9(-12) that directly ending in teeth, few glands at the leaf base and two persistent stipules at the base. Initially, we identified these plants as *Viburnum erosum*; however, considering that these plants are collected from the type locality of *V. fengyangshanense*, we compared the protologue and color plates provided by Qiu et al. [16], and found that these distinctive morphological traits also corresponded to *V. fengyangshanense*. In order to find the key morphological evidence that better distinguishes these two similar species, we futher scrutinized the type specimens, protologue and our previous collections of *V. erosum*. We believe that the discrepancies [16] between *V. fengyangshanense* (Figs. 1 and 3) and *V. erosum* (Figs. 1-2 and 4) fall within the variation range of the latter. These research results further raise an issue regarding the taxonomic relationship between *V. fengyangshanense* and *V. erosum*. To indetify the relationship between them, morphological and molecular phylogenetic studies were conducted to clarify their taxonomic status.

Material and methods

Morphological studies

We meticulously examined all the available high-resolution images of herbarium specimens of *Viburnum erosum* from A, AU, BNU, CCAU, CDBI, CSFI, CSH, FJFC, FKP, GFS, GNNU, GZTM, HNNU, HZ, IBK, IBSC, IFP, JIU, JXAU, K, KUN, L, NAS, NF, P, PE, QFNU, SDF, SZG, UPS, US, XBG, WCSBG and WUK. Since Qiu and Chen [16] did not provided any specimen images of *V. fengyangshanense*, we primarily refered to the color illustrations (Fig. 1B) and morphological description in the protologue, and conducted careful observations of *V. fengyangshanense* collected in Fengyang Mountain, Longquan County, Zhejiang, China (Type locality). All major morphological characters of *V. erosum* and *V. fengyangshanense* were studied on living plants using a digital camera (Olympus TG-6, Tokyo, Japan).

Sample collection, DNA extraction, PCR amplification, and sequencing

Based on previous phylogenetic analyses of *Viburnum* [17-21], we selected 64 samples from 11 sections in *Viburnum*, using *Sambucus canadensis* Linnaeus as the outgroup in our analysis according to the conclusions of Donoghue et al. [17]. We generated new sequences of *V. fengyangshanense, V. erosum*, *V. lancifolium* P.S. Hsu and *V. squamulosum* P.S. Hsu, while sequences for other samples were obtained from the NCBI (https://www.ncbi.nlm.nih.gov/). Voucher information and GenBank accession numbers for the materials in this study are provided in S1 Table.

Total genomic DNA of *Viburnum fengyangshanense, V. erosum*, *V. lancifolium* and *V. squamulosum* were extracted from silica gel-dried leaves using the modified CTAB method [22]. Amplification and sequencing reaction of nrITS, *mat*K, *rbc*L and *ndh*F followed Clement and Donoghue [18-19].

Phylogenetic analysis

The matrices of all sequences were aligned with MAFFT 7.450 [23]. Maximum likelihood (ML) analysis was performed using IQ-TREE [24], and bootstrap analysis was conducted with 20 000 ultrafast bootstraps. The best-fit BIC model was

determined by ModelFinder [25]. Bayesian inference (BI) was carried out by MrBayes 3.2.6 [26] with 3 000 000 generations, sampling every 1 000 generations to ensure the convergence (average deviation of split frequencies less than 0.01 and the effective sample sizes over 200). The first 25% of the sampled data were discarded as burn-in, and the remaining trees were used to estimate the posterior probabilities (PP). Bootstrap percentage (MLBS) values ≥ 70 [27] and PP values ≥ 0.90 [28] were considered strong support. Finally, the phylogenetic trees were visualized by Figtree1.4 (http://tree.bio.ed.ac.uk/software/figtree/).

Results and discussion

Morphological study

As illustrated in Figs. 1-4, the indumentum on abaxial leaves and inflorescence, the shape and size of leaves, as well as the number of lateral veins in *Viburnum erosum*, ~~exhibit~~ considerable variation. Qiu and Chen [16] pointed out that the differences in leaf size, stipule length, indumentum density on abaxial leaves and seed characteristics between *V. erosum* and *V. fengyangshanense* are crucial for their differentiation. However, based on prior descriptions [2, 4] and our morphological research findings (Figs. 1-4), there is an excessive variation in these quantitative traits, which is insufficient to distinguishing these two species, especially the seed trait, which is a significant criterion for the morphological classification of *Viburnum* plants [29-30], yet both species possess 2 shallow dorsal grooves and 3 shallow ventral grooves (Figs. 1B and 4G). Qiu and Chen [16] indicated that *V. fengyangshanense* has 5-7 lateral veins and two pairs of bud scales (a short inner layer and a long outer layer). However, the number of leaf veins in this species should be 6-11 pairs (Figs. 1B and 3), which falls within the variation range of *V. erosum* (6-14) [2, 4]. Further more, as depicted in Fig. 1A, two pairs of unequal bud scales can be clearly visible. Additionally, we also observed a few circular glands on both sides of the base on abaxial leaf (Figs. 3-4), which serve as an important taxonomic basis in *Viburnum* [20]

Phylogenetic study

Our ML and BI analyses have generated nearly identical topologies, consistent with precious results [17-21]. As shown in Fig. 5, phylogenetic results based on nrITS, *mat*K, *rbc*L and *ndh*F of 64 samples indicated that *Viburnum* forms a strongly supported clade (BS=100, PP=1.00), sect. *Odontotinus* was a monophyletic clade (BS=99, PP=1.00), and *V. fengyangshanense* clustered with two samples of *V. erosum*, forming a clade (BS=95, PP=1.00).

In summary, based on the morphological and phylogenetic results of this study, we propose that *Viburnum fengyangshanense* should be regarded as a new synonym of *V. erosum* rather than a new species.

Taxonomic treatment

Viburnum erosum Thunberg [10]; Thunberg [31], (Figs. 1A and 4).

Type:—JAPAN. Without precise locality, collection date unknown, *C. P. Thunberg s.n.* (holotype: UPS-V007398!) (Fig. 1A).

=*V. fengyangshanense* Z.H. Chen, P.L. Chiu, et L.X. Ye [16], ***syn. nov.*** (Figs. 1B and 3).

Type:—CHINA. Zhejiang, Longquan, Fengyang Moutain, Fengyang Lake, bush and slope, 1570 m a.s.l., 8 May 2016, *Z. H. Chen, L. X. Ye & S. L. Liu LQ2016001* (holotype: ZM; isotype: HTZ, ZM).

=*V. erosum* var. *atratocarpum* Hsu [11].

Type:—CHINA. Shanxi, Liuba, Huoshaodian Village, slope and forest margins, 1050 m a.s.l., 15 October 1952, *F. K. Jun 6222* (isotype: IBK00021901!) (Fig. 2A).

=*V. erosum* var. *ichangense* Hemsley [13].

Type:—CHINA. Hubei, Ichang (Yichang) Patung District, March 1886, *A. Henry 232* (syntype: K000797962!) (Fig. 2B); February 1887, *A. Henry 2841* (syntype: K000797964!); Hubei, Ichang February 1887, *A. Henry 1888* (syntype: K000797965!).

=*V. erosum* var. *setchuenense* Graebner [14].

Type:—CHINA. Sichuan, without precise locality, 1900, *C. Bock & A. V. Rosthorn 2297*

(isotype: A00031556!) (Fig. 2C).

=*V. taquetii* H. Léveillé [15].

Type:—SOUTH KOREA. Sylvis Yengsil, Quelpaert, 1000 m a.s.l., 12 August 1910, *T. Taquet 4281* (holotype: E00265361!; isotype: A00031557!) (Fig. 2D).

The detailed morphological description refers to Hsu [4], and Yang & Malécot [2].

Distribution and habit:—*Viburnum erosum* is widely distributed in northern China, southern China, southeastern China, Japan, and Korea (Fig. 6). It grows in forests and shrubs at elevations of 300–2300 m.

Phenology:—Flowering April ~~to~~ May; Fruiting August to October.

Additional specimens examined:—**CHINA. Anhui,** Huoshan, *M. B. Deng & Z. G. Wei 80166* (NAS00266165); Jinzhai, *K. Yao 9901* (NAS00614827); Qianshan, *X. H. Shi 39* (NF2015928); Shitai, *R. P. Jiang et al. 940* (NAS00614815); Yuexi, *Z. W. Xie & L. Zheng 97298* (PE02115525). **Chongqing,** *L. Q. Gao s.n.* (JXAU). **Fujian,** Zhangzhou, Nanjing, *T. P. Zhu 1233* (AU066161); Longhai, *C. M. Tan 971606* (SZG00044688); Nanping, *S. S. You 989* (FJFC0002625). **Gansu,** Chengxian, *Z. P. Wei 2227* (WUK0108949); Kangxian, *Y. Q. He & C. L. Tang 223* (WUK0215012); Wenxian, *Z. X. Hu 689* (IBSC0542031). **Guangdong,** Heping, *G. C. Zhang 96* (IBSC0541699); Renhua, *L. Tang 7443* (IBSC0541726); Shixin, *L. Tang 7132* (IBSC0541725); Wengyuan *X. Q. Liu 23929* (IBK00020993). **Guangxi,** Guanyang, *Z. Z. Chen 52136* (IBSC0541731); Pinggui, Y. Liu et al. *H0174* (IBK00323438); Quanzhou, *Z. S. Chung 81570* (IBSC0541730); Rongshui, *S. Q. Chen 16447* (PE0541728); Xingan, *L. G. Zhao & B. Lu 63203*, (IBK00189025). **Guizhou,** Dafang, *R. B. Jiang 705* (IBSC0542019); Leishan, *Y. F. Li 522634150830007LY* (GZTM0063374); Wengan, *J. B. Zuo D-370* (GFS0013538); Xingren, *Y. T. Zhang 7341* (IBSC0542021). **Henan,** Shangcheng, *C. S. Zhu et al. 13080835* (AU067206); Xinyang, *Plant Resource Investigation Team D0728* (PE01130026). **Hubei,** Baokang, *C. Q. Wang 3-001* (CCAU0007423); Hefeng, *H 1743* (CCAU0007856); Xianfeng, *W. B. Lin 512* (WUK0310836); Xuanen, *H. C. Li 3839* (IBSC0541876); Zhuxi, *P. Y. Li 9271* (WUK0356317). **Hunan,** Cili, G. B. Zhu 43 (IBSC0541840); Hengshan, H. T. Chang 3040 (IBSC0541864); Linwu, *L. Ang LA0144* (CSFI064335); Luxi, *D. G.*

Zhang 4331221607170767LY (JIU31158); Yizhang, *W. J. Liu & X. F. Song* (CSFI075586); Yuanling, G. C. Zhang 360 (PE01130597). **Jiangsu,** Danyang, *Nanlin Team 7250* (IBSC0541990); Wuxi, *G. Yao et al. 10687* (NAS00138216); Yixing, *G. Yao 7071* (NAS00138215). **Jiangxi,** Chongyi, *Y. Y. Cong 33398* (HNNU00062513); Jinggangshan, *Y. L. Liu 46* (JXAU); Jiujiang, *Y. L. Liu 31* (JXAU); Xunwu, *Z. B. Tang* (GNNU0006512); Yichun, *H. G. Ye & F. Y. Zeng LXP 10-2435* (IBSC0775645), *X. H. Wu et al. 2* (JXAU). **Shandong,** Maoping, *L. Q. Qiu 4211* (WUK0312333); Qingdao, *T. Y. Zhou 1188* (NAS00266265); Yantai, *Y. T. Hou & C. Y. Guo* 2014028 (QFNU0015829). **Shannxi,** Langao, *P. Y. Li 8470* (WUK0355776); Lueyang, *K. J. Fu 6016* (IBK00334551); Taibai, *K. J. Fu 5066* (WUK0059531); Zhenping, *G. Y. Xu 4910* (WUK0475774). **Sichuan,** Maoxian, *X. Y. Xiong et al. 2004005* (WCSBG007033); Dayi, *H. Hai, Y. K. Li & Y. P. Du DY0469* (CDBI028543); Nanjiang, *Y. He & H. Tian GWS20154161* (BNU0019539); Tongjiang, *Bashan Collection Team 5862* (PE02066229); **Taiwan**, Hualien, *R. Q. Gao et al. 1256* (KUN760743); Nantou, *J. G. Liu 995* (PE01130764); Taichung, *T. C. Chen 11881* (PE01775364), *T. C. Chen 11880* (PE00869145), *12116* (PE00869146), *M. J. Liu 12* (PE01646064); Taipei, Datong, *T. Y. Liu 933* (PE01446679); Yilan, *Z. Y. Wang & J. G. Liu 43* (PE01130767, PE01130766), *S. Y. Lv s.n.* (PE01130768). **Yunnan,** Shuifu, *W. G. Yang DT6662* (KUN15130048); Suijiang, *Z. H. Hu 1366* (IBSC0541733); Yiliang, *Z. Z. Ding & J. X. Wang 1091* (NAS00266608); Zhenxiong, *S. Y. Bao 132* (KUN85618). **Zhejiang,** Anji, *H. X. Yu 24232* (HZ054851); Kaihua, *H. X. Yu 29711* (IBSC0541965); Qingyuan, *S. Y. Zhang 3472* (NAS00266285); Suichang, *Q. F. Zhang et al. ZX00213* (CSH0121231); Taishun, *S. Y. Zhang 3735* (HZ055065).

JAPAN. Nagasaki, precise locality unknown, *C. L. Maximowicz s.n.* (L2974728, L2974729), (US 97369, US 97370); *R. Oldham 477* (L2974727); **Yokoska**, Nakatosa town, *T. Miyazaki 1405054* (P01168582); precise locality unknown, *H. Savatier 536* (US 97373), *M. Labbé 70* (P03318885), *363* (P03318884), *4154* (P03318879), *6185* (P03318880), *M. Labbé s.n.* (P03318881).

NORTH KOREA. Hwanghae-namdo, precise locality unknown, *U. J. Faurie 680* (FKP86519); **Northern Gangwon-do**, precise locality unknown, *S. E. Liu 9398*

(IFP13606999y0017), *G. Koidzumi s.n.* (FKP23836), *E. H. Wilson 10401* (FKP78065).

SOUTH KOREA. Gangwon-do, precise locality unknown, *K. Kondo 9113* (PE01618021); Yangyang-gin, *K. Y. Chul KYC2007* (KUN1224955); **Chol-la-nam-do**, precise locality unknown, *W. H. Takahashi & C. S. Chang 344* (PE01618015), *C. S. Chang 1167* (PE01618016), *W. H. Takahashi & C. S. Chang 378* (PE01618014); **Incheon**, Ganghwa, *H. R. Na, K. Yoo & H. S. Kim N-EX11* (PE02112636); **Jeju-do**, Seogwipo, Jungmun, *B. U. Oh et al. 120612-006* (PE01920990); **Jeollanam-do**, Gangjin, *H. S. Jung 201128-0046* (PE02040482).

Supporting information

S1 Table. Voucher information and GenBank accession numbers for the materials in this study. Star symbol indicates the newly obtained sequences.

Acknowledgements

We are grateful to the curators of the herbaria of A, AU, BNU, CCAU, CDBI, CSFI, CSH, FJFC, FKP, GFS, GNNU, GZTM, HNNU, HZ, IBK, IBSC, IFP, JIU, JXAU, K, KUN, L, NAS, NF, P, PE, QFNU, SDF, SZG, UPS, US, XBG, WCSBG and WUK for allowing us to examine and use their scanned images of specimens, especially for UPS providing the holotype of *Viburnum erosum*, where further promote our research. In addition, we would like to express gratitude to researcher Ren Chen of the South China National Botanical Garden of the Chinese Academy of Sciences for his constructive guidance. This work was supported by the National Natural Science Foundation of China (grant no. 31960043).

References

1. Linnaeus C. *Species Plantarum*, vol. 1. Laurentius Salvius, Stockholm.1753; 267 pp.
2. Yang QE, Malécot V. *Viburnum* Linnaeus. In: Wu ZY, Raven PH, Hong DY (Eds.) Flora of China, vol. 19. Science Press, Beijing & Missouri Botanical Garden Press, St. Louis. 2011; pp. 570−611.
3. Cronquist A. Evolution and Classification of Flowering Plants, vol. 2. The New York Botanical Garden, New York. 1988; pp. 513−517.
4. Hsu PS. *Viburnum* Linnaeus. In: Hsu, P.S. (Ed.) Flora Reipublicae Popularis Sinicae, vol. 72. Science Press, Beijing. 1988; pp. 12−104. [In Chinese]
5. Backlund A, Bremer B. Phylogeny of the Asteridae s. str. based on *rbcL* sequences with particular reference to the Dipsacales. Plant Systematics and Evolution. 1997; 207: 225–254. https://doi.org/10.1007/BF00984390
6. Angiosperm Phylogeny Group. An update of the angiosperm phylogeny group classification for the orders and families of flowering plants: APG III. Botanical Journal of the Linnean Society. 2009; 161(2): 105–121. http://dx.doi.org/10.1111/j.1095-8339.2009.00996.x
7. Angiosperm Phylogeny Group. An update of the Angiosperm Phylogeny Group classification for the orders and families of flowering plants: APG IV. Botanical Journal of the Linnean Society. 2016; 181(1): 1–20. http://dx.doi.org/10.1111/boj.12385
8. Reveal JL. (1800–1802) Proposals to conserve the name Viburnaceae (Magnoliophyta), the name Adoxaceae against Viburnaceae, a "superconservation" proposal, and, as an alternative, the name Sambucaceae. Taxon. 2008; 57(1): 303. https://doi.org/10.2307/25065975
9. Turland NJ, Wiersema JH, Barrie FR, Greuter W, Hawksworth DL, Herendeen PS, et al. (Eds.) International Code of Nomenclature for algae, fungi, and plants (Shenzhen Code) adopted by the Nineteenth International Botanical Congress Shenzhen, China, July 2017. Regnum Vegetabile. 2018; 159. Glashütten: Koeltz

Botanical Books. https://doi.org/10.12705/Code.2018

10. Murray JA. Systema Vegetabilium, vol. 14. Göttingen. 1784; 295 pp.
11. Hsu PS. Notes on genus *Viburnum* of China. Acta Phytotaxonomica Sinica. 1975; 13 (1): 111–128. [In Chinese]
12. Qiu BL. Florula of genus *Viburnum* Linn. in Zhejiang. Journal of Zhejiang Forestry College.1988; 5(2): 133–144. [In Chinese]
13. Forbes FB, Hemsley WB. Enumeration of all the plants known from China proper, Formosa, Hainan, the Corea, the Luchu Archipelago, and the island of Hongkong: Together with their distribution and synonymy. Linnean Society. 1888; 23: 352.
14. Engler A. Botanische Jahrbücher für Systematik, Pflanzengeschichte und Pflanzengeographie, vol. 29. Leipzig, Engelmann. 1901; 589 pp.
15. Léveillé H. Decades plantarum novarum. LIV–LVIII. Repertorium novarum specierum regni vegetabilis, vol. 9. Berlin, Selbstverlag des Herausgebers.1911; pp. 443−444.
16. Qiu BL, Ye LX, Chen F, Xie WY, Chen ZH. Additional notes on *Viburnum* L. in Zhejiang. Journal of Hangzhou Normal University. 2020; 19 (3): 261−266. [In Chinese] https://doi.org/10.12191/j.issn.1674−232X.2020.03.008
17. Donoghue MJ, Baldwin BG, Li J, Winkworth RC. *Viburnum* phylogeny based on chloroplast *trnK* intron and nuclear ribosomal ITS DNA sequences. Systematic Botany. 2004; 29(1): 188−198. https://doi.org/10.1600/036364404772974095
18. Clement WL, Donoghue MJ. Dissolution of *Viburnum* section *Megalotinus* (Adoxaceae) of Southeast Asia and its implications for morphological evolution and biogeography. International Journal of Plant Sciences. 2011; 172(4): 559−573. https://doi.org/10.1086/658927
19. Clement WL, Donoghue MJ. Barcoding success as a function of phylogenetic relatedness in *Viburnum*, a clade of woody angiosperms. BMC evolutionary biology. 2012; 12(1): 1−13. https://doi.org/10.1186/1471-2148-12-73
20. Clement WL, Arakaki M, Sweeney PW, Edwards EJ, Donoghue MJ. A chloroplast tree for *Viburnum* (Adoxaceae) and its implications for phylogenetic classification and character evolution. American Journal of Botany. 2014; 101(6):

1029−1049. https://doi.org/10.3732/ajb.1400015

21. Landis MJ, Eato DAR, Clement WL, Park B, Spriggs EL, Sweeney PW et al. Joint phylogenetic estimation of geographic movements and biome shifts during the global diversification of *Viburnum*. Systematic Biology. 2021; 70(1): 67–85. https://doi.org/10.1093/sysbio/syaa027
22. Doyle JJ, Doyle JL. A rapid DNA isolation procedure for small quantities of fresh leaf tissue. Phytochemical Bulletin. 1987; 19: 11–15.
23. Katoh K, Standley DM. MAFFT multiple sequence alignment software version 7: Improvements in performance and usability. Molecular Biology and Evolution. 2013; 30(4): 772–780. https://doi.org/10.1093/molbev/mst010
24. Nguyen LT, Schmidt HA, Von Haeseler A, Minh BQ. IQ-TREE: A fast and effec tive stochastic algorithm for estimating maximum-likelihood phylogenies. Molecular Biology and Evolution.2015; 32(1): 268–274. https://doi.org/10.1093/molbev/msu300
25. Kalyaanamoorthy S, Minh B, Wong T, von Haeseler A, Jermiin L. ModelFinder: Fast model selection for accurate phylogenetic estimates. Nature Methods. 2017; 14(6): 587–589. https://doi.org/10.1038/nmeth.4285
26. Ronquist F, Teslenko M, van der Mark P, Ayres DL, Darling A, Höhna S. et al. MrBayes 3.2: Efficient Bayesian phylogenetic inference and model choice across a large model space. Systematic Biology. 2012; 61(3): 539–542.
27. Suzuki Y, Glazko GV, Nei M. Overcredibility of molecular phylogenies obtained by Bayesian phylogenetics. Proceedings of the National Academy of Sciences. 2002; 99(25): 16138−16138. DOI:10.1073/pnas.212646199.
28. Jacobs B, Donoghue MJ, Bouman F, Huysmans S, Smets E. Evolution and phylogenetic importance of endocarp and seed characters in *Viburnum* (Adoxaceae). International journal of plant sciences. 2008; 69(3): 409–431. https://doi.org/10.1086/tax.526468
29. Jacobs B, Huysmans S, Smets E. Evolution and systematic value of fruit and seed characters in Adoxaceae (Dipsacales). Taxon. 2010; 59(3): 850–866. https://doi.org/10.1002/tax.593014

30. Thunberg CP. Flora Japonica: sistens plantas insvlarvm Japonicarvm secvndvm systema sexvale emendatvm, vol. 1. Lipsiae, Mulleriano. 1784; 124 PP.

 Click here to access/download;Figure;Fig1.tiff

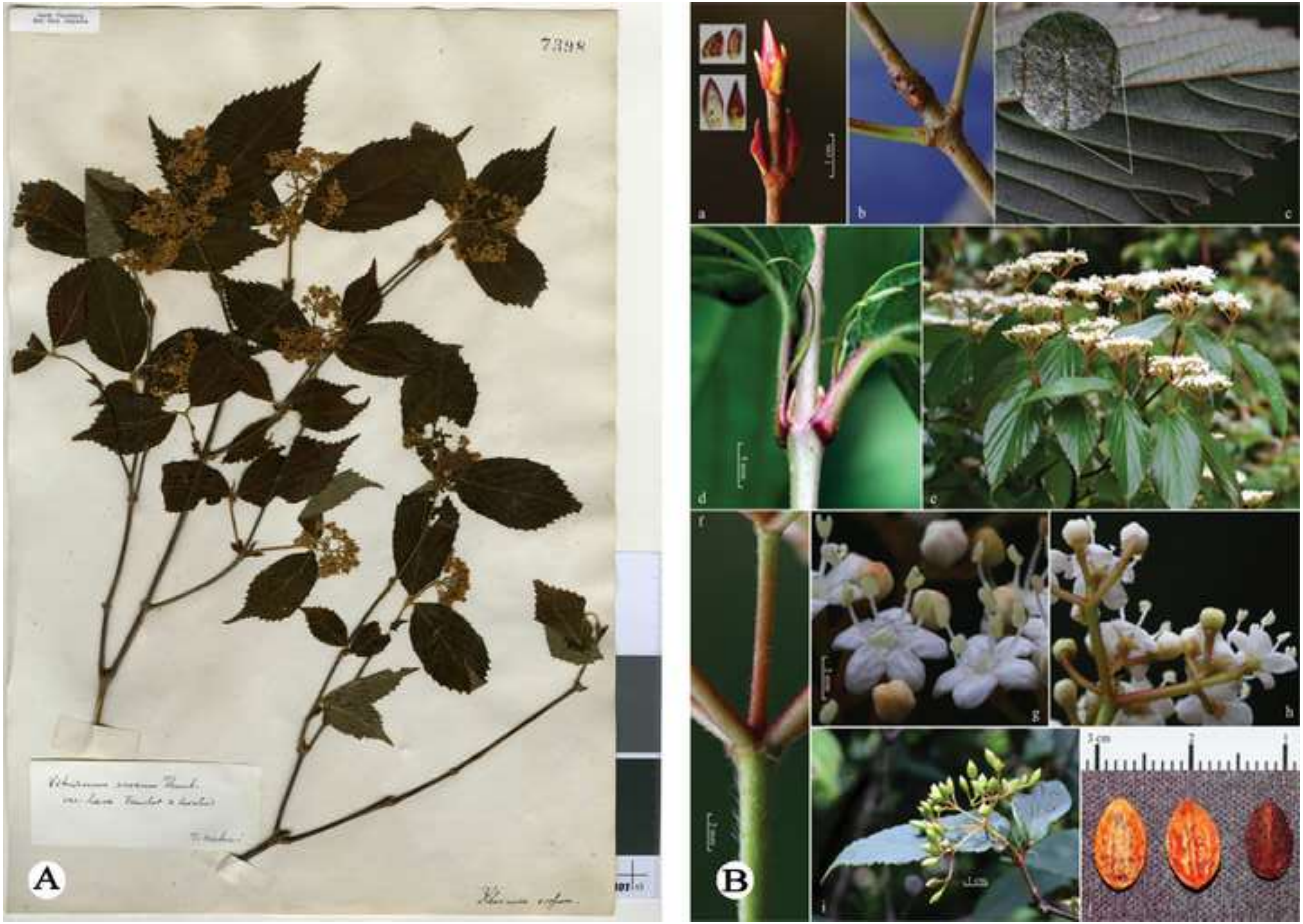

Click here to access/download;Figure;Fig2.tiff

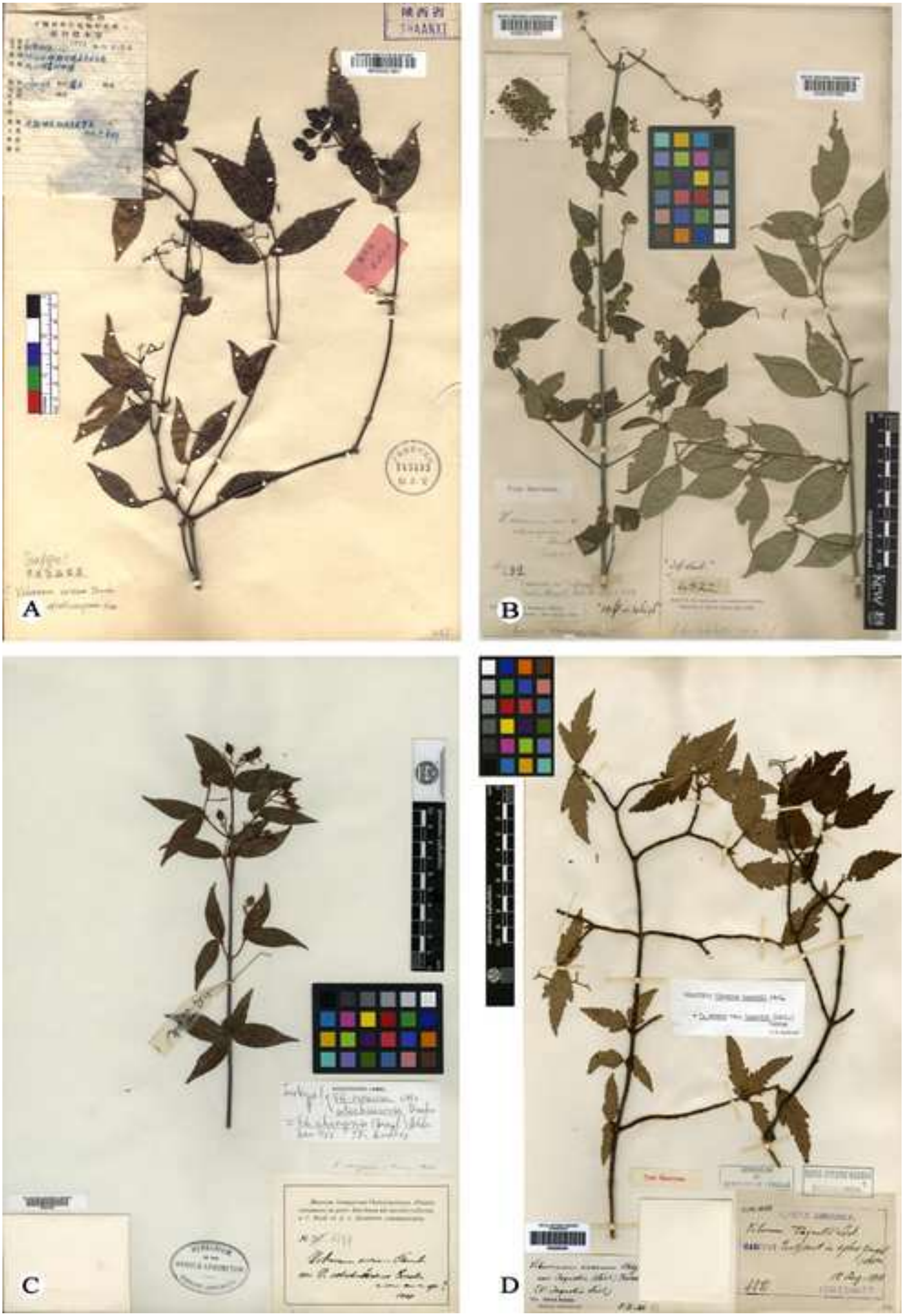

Morphology of Viburnum fengyangshanense (= V. erosum)

Click here to access/download;Figure;Fig3.tiff

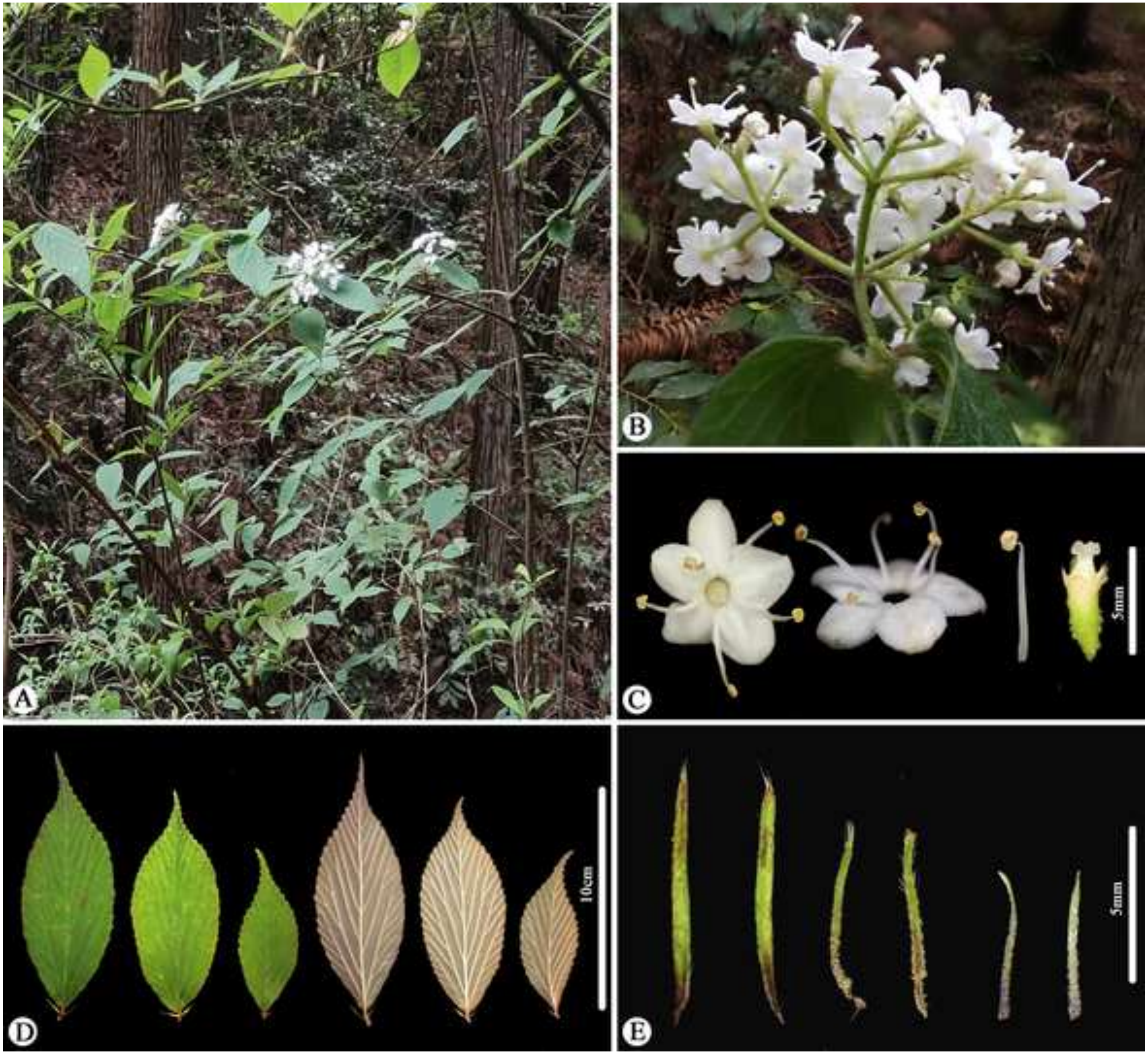

Morphology of Viburnum erosum

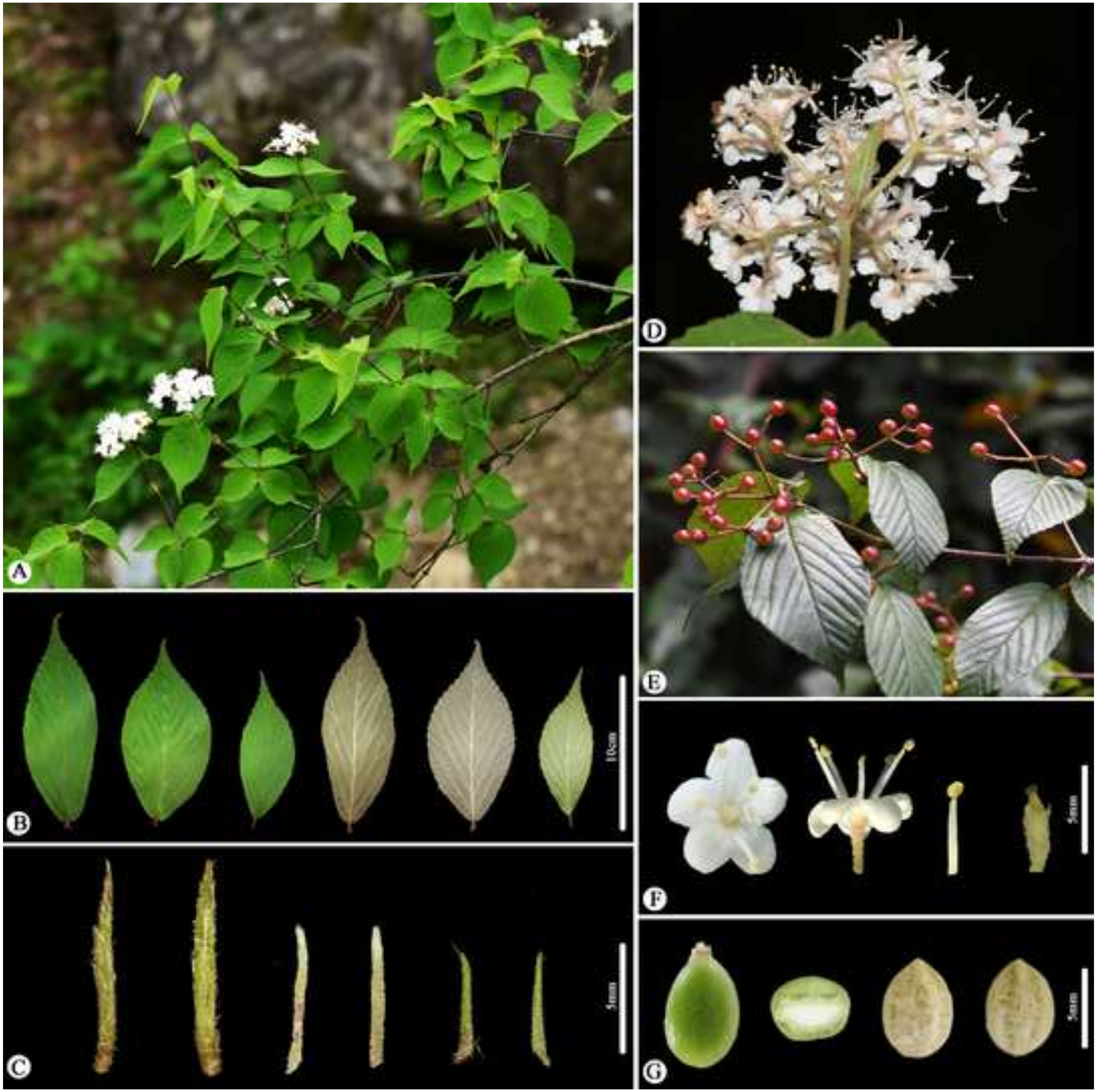

The phylogenetic tree

Click here to access/download;Figure;Fig5.tif

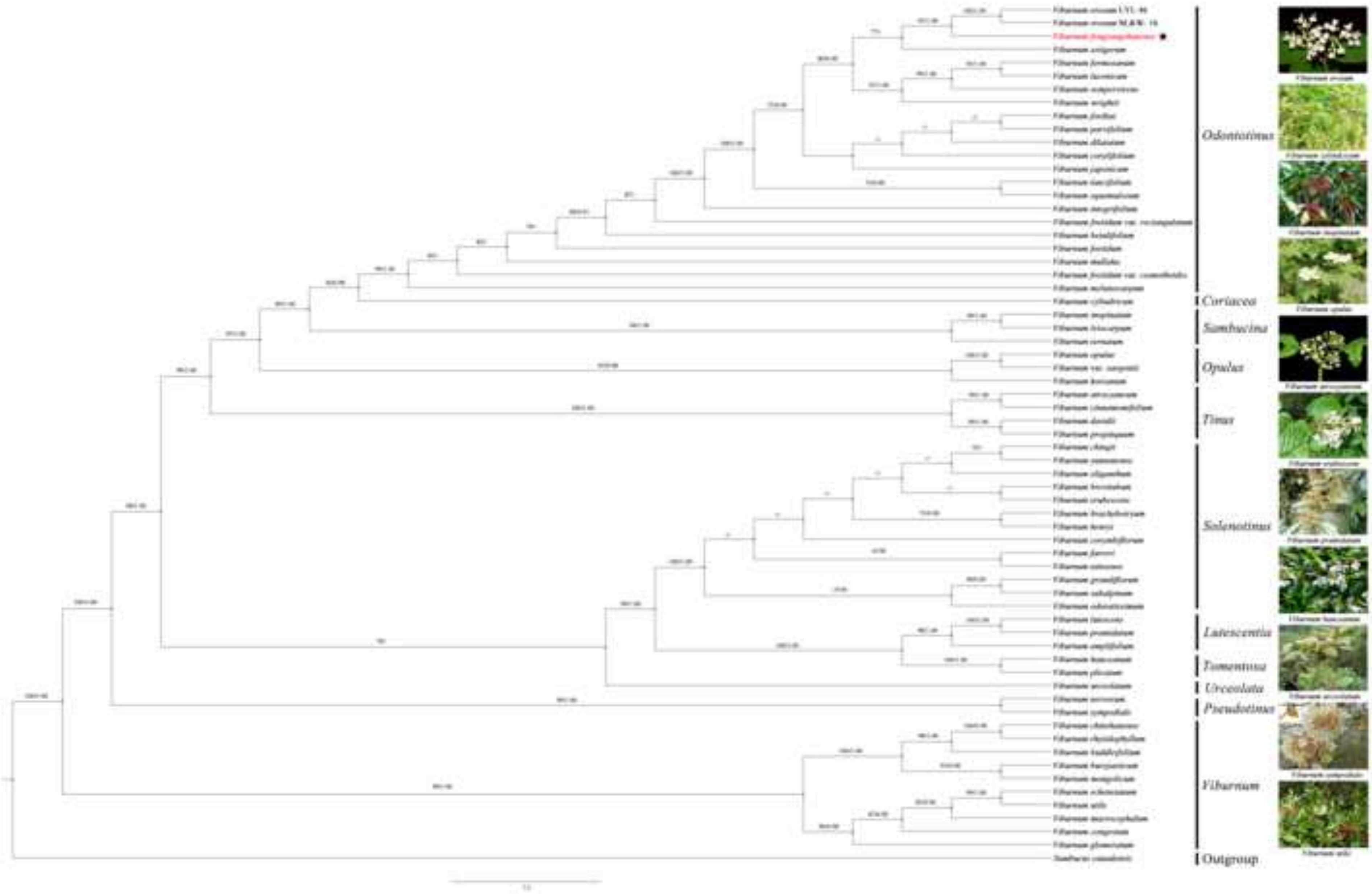

Geographical distribution of Viburnum erosum

Click here to access/download;Figure;Fig6.tif

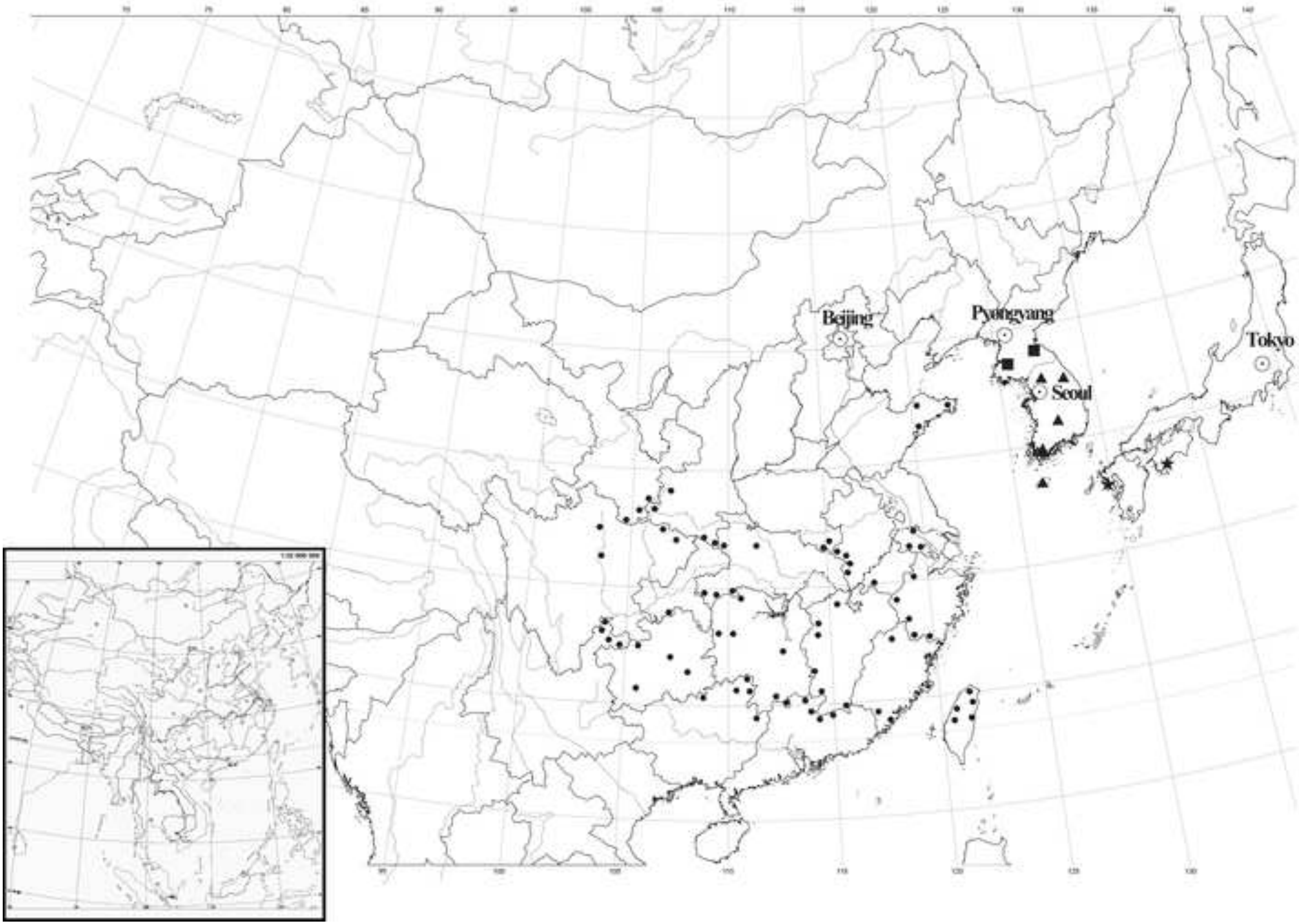

Supplement: Attachment — Submitted filename: PONE-D-24-45964_reviewer.pdf [file pone.0312920.s002.pdf]
