# Supplementary material for: A new synonym for Viburnum erosum (Viburnaceae) in East China, based on morphological and molecular evidence
Source: PLoS One. 2025 Apr 23;20(4):e0312920. doi: 10.1371/journal.pone.0312920 (PMC12017528; doi:10.1371/journal.pone.0312920)
Supplement: Attachment — Submitted filename: PONE-D-24-45964.pdf [file pone.0312920.s003.pdf]

Dear editor

I am writing to provide my review of the manuscript titled **"A new synonym for *Viburnum erosum* (Viburnaceae) in East China, based on morphological and molecular evidence"** (Manuscript Number: PONE-D-24-45964), submitted to PLOS ONE.

Summary:

This study combines morphological and molecular evidence to synonymize *Viburnum fengyangshanense* with *V. erosum*. The methodology is robust, using nrITS and plastid DNA markers (*rbcL*, *matK*, *ndhF*), along with detailed morphological analyses.

Strengths:

- Integration of molecular and morphological data.
- Well-structured manuscript with clear figures.
- Valuable contribution to *Viburnum* taxonomy.

Weaknesses:

1. Limited molecular sampling reduces the generality of conclusions.
2. Insufficient discussion on ecological and conservation implications.
3. Methodological details, especially bioinformatics, need clarification.
4. Limited comparison with prior phylogenetic studies.

Recommendation:

With minor revisions addressing these weaknesses, the manuscript has the potential for publication. The study aligns with the interdisciplinary scope of PLOS ONE and contributes valuable taxonomic insights. The decision for acceptance rests with the editorial team.

Thank you for the opportunity to review this manuscript.

Sincerely

Ali
