# Supplementary material for: A new synonym for Viburnum erosum (Viburnaceae) in East China, based on morphological and molecular evidence
Source: PLoS One. 2025 Apr 23;20(4):e0312920. doi: 10.1371/journal.pone.0312920 (PMC12017528; doi:10.1371/journal.pone.0312920)
Supplement: Attachment — Submitted filename: Response to Reviewers.docx [file pone.0312920.s005.docx]

15 February 2025

Dear Reviewers,

We sincerely appreciate your insightful comments and constructive suggestions, which have significantly improved the quality of our manuscript. We have carefully addressed all the points raised in your review, and the revised manuscript now incorporates these changes. A clean version of the manuscript, along with a version highlighting all modifications (**in red**), has been uploaded for your convenience. Below, we provide a point-by-point response to your comments, detailing the specific revisions made.

Once again, we thank you for your time and expertise in evaluating our work. We believe the manuscript has been substantially strengthened through this revision process, and we look forward to your further feedback.

Yours sincerely,
Ming Tang
On behalf of all authors

**Response to Anonymous Reviewer 1:**

Comment 1: Addition of references for *Adoxa*, *Tetradoxa* and *Sinadoxa*.
**Response:**
References for *Adoxa* Linnaeus [6], *Tetradoxa* C.Y. Wu [7], and *Sinadoxa* C.Y. Wu, Z.L. Wu & R.F. Huang [8] have been integrated into the reference section.

Comment 2: Clarification on the nomenclature of Viburnaceae.
**Response:**
The taxonomic rationale for retaining Viburnaceae has been revised to align with the International Code of Nomenclature (ICN) guidelines (Lines 42–45). Specifically, we emphasize that Viburnaceae was conserved under ICN Article 14.10, whereas the proposal to "superconserve" Adoxaceae was rejected, as per the rulings of the Nomenclature Committee for Vascular Plants.

Comment 3: Are such characteristics: leaf size, stipule length, indumentum density on abaxial leaves and seed, commonly used to recognize species in this genera?

**Response:** These morphological features are widely recognized as critical taxonomic criteria for identify *Viburnum* sect. *Odontotinus* [2,4], with both *V. erosum* and *V. fengyangshanense* being taxonomically placed within this section.

**Response to Anonymous Reviewer 2:**

**Weakness 1:** Limited molecular sampling reduces the generality of conclusions.

**Response:** In this study, we conducted comprehensive sampling of 64 samples representing 61 species and 3 varieties across 11 taxonomic sections within *Viburnum*, encompassing 83.4% (73 species) of the total Viburnum species distributed in China. Notably, sect. *Odontotinus* was particularly well-represented, with 19 species and 2 varieties sampled, accounting for 86.4% (22 species) of its Chinese distribution. This extensive sampling strategy provides a robust framework for reconstructing the phylogenetic relationships of Chinese *Viburnum* and offers critical evidence for resolving the phylogenetic placements of *V. erosum* and *V. fengyangshanense*.

**Weakness 2:** Insufficient discussion on ecological and conservation implications.

**Response:** A new subsection “Conservation Implications” has been added (Line 175-197 in “Revised Manuscript with Track Changes.docx”)

**Weakness 3:** Methodological details, especially bioinformatics, need clarification.

**Response:** The Materials and Methods section now details:

In this study, we employed second-generation high-throughput sequencing to generate complete chloroplast genomes and nuclear ribosomal DNA (nrDNA) assemblies for four *Viburnum* taxa: *V. erosum*, *V. fengyangshanense*, *V*. *lancifolium*, and *V. squamulosum*. Three plastid markers (*matK*, *rbcL*, and *ndhF*) were extracted from the chloroplast genomes using Geneious Prime software, while nrITS sequences were isolated and curated with ITSx. Although whole chloroplast genome data were generated, phylogenetic analyses focused on these three plastid loci combined with nrITS due to the limited availability of complete chloroplast genome sequences for Chinese *Viburnum* species. This marker combination strategically targets evolutionarily informative regions that are widely represented in existing datasets, enabling comprehensive phylogenetic comparisons across Chinese *Viburnum* lineages. (Line 110-127 in “Revised Manuscript with Track Changes.docx”)

**Weakness 4:** Limited comparison with prior phylogenetic studies

**Response:** The primary objective of this study is to elucidate the phylogenetic relationship between*V. erosum* and *V. fengyangshanense* through integrated morphological and molecular evidence. Our findings robustly support the monophyly of the *Viburnum* and the sect. *Odontotinus* (BS = 98, PP = 1.00). Notably, the samples of *V. fengyangshanense* form a well-supported clade (BS = 95, PP = 0.99) with two accessions of *V. erosum,* which providing conclusive evidence to resolve the phylogenetic placement of these taxa. These results not only achieve the primary aim of this study but also offer new insights into the evolutionary history of the *Viburnum* lineage in East Asia. (Line 162-171 in “Revised Manuscript with Track Changes.docx”)
